# Indeterminacy tolerance as a basis of hemispheric asymmetry within prefrontal cortex

**DOI:** 10.3389/fnhum.2015.00326

**Published:** 2015-06-16

**Authors:** Vinod Goel

**Affiliations:** ^1^Department of Psychology, York UniversityToronto, ON, Canada; ^2^IRCCS Fondazione Ospedale San CamilloVenice, Italy

**Keywords:** hemispheric asymmetry, hemispheric specialization, reasoning, hemispheric lateralization, real-world problem solving, ill-structured problems

## Abstract

There is an important hemispheric distinction in the functional organization of prefrontal cortex (PFC) that has not been fully recognized and explored. Research with split-brain patients provides considerable evidence for a left hemisphere (LH) “interpreter” that abhors indeterminacy and automatically draws inferences to complete patterns (real or imaginary). It is suggested that this “interpreter” function may be a byproduct of the linguistic capabilities of the LH. This same literature initially limited the role of the right hemisphere (RH) to little more than visual organization. Recent reviews have garnered evidence for several different roles for the right PFC in reasoning, problem solving, and decision-making. We here focus on the beneficial but neglected role of indeterminacy in real-world problem solving and argue that the right PFC complements the left PFC “interpreter” by maintaining, and even enhancing indeterminacy. Successful real-world functioning is a delicate balancing act between these two systems.

## Introduction

Some degree of hemispheric asymmetry seems to be a principle of brain organization in most, if not all, species (Denenberg, 1981; Toga and Thompson, 2003). Since the issue came to prominence in the 1950s, with the pioneering work by Sperry and colleagues (Sperry, 1982; Gazzaniga, 1995), on split brain patients, a number of studies have demonstrated differences in hemispheric organization ranging all the way from physiological and structural cellular organization (Glick et al., 1982; Zilles et al., 1996), to functional differences at the level of sensory motor (Amunts et al., 1996; Coghill et al., 2001), language (Levy et al., 1971; Levy, 1976; Knecht et al., 2000), visual-spatial (Ratcliff, 1979; Christman, 1989), attention (Corbetta and Shulman, 2002), emotion (Davidson, 1992), and complex cognition systems (Gazzaniga, 1985, 1995).

Despite overwhelming evidence for hemispheric asymmetry, answers to questions regarding the functional basis of the asymmetry remain elusive (Springer and Deutsch, 1998). Proposals fall into two basic categories: they either focus on the properties of the stimuli, or characterize asymmetry in terms of differences in processing styles (Stephan et al., 2003). With respect to the former, the distinction has been between verbal and visual-spatial modalities, where verbal is largely left hemisphere (LH) and visual-spatial is largely right hemisphere (RH; Ratcliff, 1979; Frost et al., 1999). Proposals that implicate different processing styles include distinctions between analytic and sequential processing (LH dominance) and holistic and parallel processing (RH dominance; Deglin and Kinsbourne, 1996; Wharton and Grafman, 1998), the spatial frequency hypothesis which distinguishes between local information processing (LH dominance) and global information processing (RH dominance; Sergent, 1987; Robertson and Lamb, 1991), and categorical (LH dominance) and coordinate (RH dominance) representation of spatial information (Kosslyn et al., 1989; Jager and Postma, 2003).

While each proposal captures some aspect of the data, none has been successful in explaining a wide range of findings. For example, the analytic/holistic distinction predicts that deductive reasoning should be a largely LH process, while inductive reasoning should be largely a RH process. The data indicate otherwise (see below). It is possible that we are making a mistake in searching for a single basis for the asymmetry and assuming that it applies equally to all brain regions and cognitive processes. Perhaps there are multiple bases of asymmetry and different brain regions rely upon different ones (or even multiple ones). The goal of this review is to introduce the principle of indeterminacy and suggest that it plays an important role in the hemispheric organization of prefrontal cortex (PFC) in complex cognition.

I use the term “indeterminacy” to refer to uncertainty independent of risk/reward evaluations, where, the issue is not that a certain event will occur with a certain probability *p* = x, but there is literally no fact of the matter. For example, given the premises A > B, A > C, what is the relationship between B and C? There are no probabilities to be assigned here. Given this incomplete information, there is no fact of the matter as to the relationship between B and C. It is indeterminate.^1^ The main thrust of the argument is that there are hemispheric differences in the ability of PFC to tolerate indeterminacy. The left PFC does not tolerate indeterminacy well and will always attempt to fill in with background information. It is suggested that this may be a consequence of the LH’s dominance for language. The right PFC is very good at tolerating, and even actively maintaining and enhancing indeterminacy and keeping options open. Both systems are required for successful problem-solving in the real world.

## Asymmetry and Complex Cognition: Left Hemisphere Interpreter Hypothesis

Perhaps the most interesting work in the asymmetric organization of the human brain for complex cognition has been undertaken by Gazzaniga and colleagues (Gazzaniga, 1995, 1998). In one classic experiment involving implicit inference, split brain patients were presented with a picture of a chicken claw projected to the LH (right visual field) and a picture of a snowy winter scene projected to the RH (left visual field). The patient must then select (one with each hand), from an array of other pictures, which two are related to the projected pictures. The patient selects a shovel with the left hand (because the right-hemisphere, controlling that hand, has viewed a snowy winter scene) and a chicken with the right-hand (because the LH, controlling that hand, viewed the chicken claw). Upon being asked to explain the choice of the shovel with the left hand (guided by the RH) the patient’s LH (dominant for language) has no access to the information about the snowy scene viewed by the RH. But instead of responding “I don’t know”, he fabricates a plausible story, based upon background knowledge, and responds that the shovel is required to clean the chicken coop.

In another simpler paradigm, a picture of a saucepan, followed by a picture of water, is shown to each hemisphere, (Gazzaniga and Smylie, 1984). When the pictures are shown to the LH, the patient can draw the causal inference of “boiling water”. When the pictures are shown to the RH, the patient cannot draw the inference. Such findings have led to the postulation of the LH “interpreter”, (Gazzaniga, 1995; Wolford et al., 2000; see also Thompson-Schill et al., 1997; Hagoort, 2005, for related ideas) a system compelled to connect bits of incomplete information to make sense of the world by locking onto and extrapolating patterns (logical, conceptual, causal, etc.). It abhors uncertainty, and automatically fills in any gaps with assumptions based upon background knowledge and beliefs, often prematurely and incorrectly. This has resulted in a story of LH dominance for reasoning and problem-solving. More recently these claims have been localized to PFC (Wolford et al., 2000) and the components of the interpreter are being fleshed out.

Marinsek et al. (2014) in a review, in this volume, highlight three functions of the left PFC in reasoning and problem-solving: (a) reduction of uncertainty; (b) sensitivity to causal connections and inferences; and (c) inferences involving logical relationships. To this list I would add inferences involving conceptual/semantic relationships (if not already included). While my focus here is largely limited to the left PFC’s role in the reduction of uncertainty or indeterminacy, I will suggest that the latter properties may help account for the former, and that all may be a byproduct of the linguistic ability of the LH. Let’s look at this by examining the role of the left PFC in deductive reasoning.

Deductive reasoning tasks involving syllogisms engage left PFC when arguments contain believable content (e.g., all apples are red fruit; all red fruit are delicious; therefore apples are delicious) allowing subjects to draw upon conceptual and semantic connections, but engage bilateral frontal systems when arguments do not contain meaningful content (e.g., all A are B; all B are C; therefore all A are C) and formal methods must be utilized. In the former case, both the semantic information and logical connections lead to the correct inference and the LH is often sufficient. In the latter case, there is no semantic information, and no substitute for explicit formal logical evaluation. In such cases the RH is recruited to assist in the inference (Goel et al., 2000, 2004).^2^ That the left PFC is primarily drawing upon conceptual and semantic connections is consistent with the results of the above experiments involving the “chicken coop” and “boiling water” tasks. For example, there are no logical connections between “saucepan” and “water”, leading to the conclusion of “boiling water”, but given our world knowledge and beliefs, there are conceptual connections. Furthermore, when the believability of the conclusion is inconsistent with the logical evaluation, and an effortful, explicit logical evaluation is required, the engagement of right PFC and parietal lobes becomes necessary (Goel et al., 2000; Goel and Dolan, 2003; Tsujii et al., 2010, 2011).

These and other data suggest that in logical inference the left PFC interpreter is usually our first response mechanism and when conceptual connections and simple logical relations are involved, it is sometimes sufficient. Several linguists have argued that our conceptual and logical (and causal) constructs and relationships are built into the very fabric of natural language and derive from it (Talmy, 1983, 1988, 2003; Lakoff, 1986). Thus it is possible that the causal, logical, and conceptual inferences that come so effortlessly to the LH do so because it is dominant for language. However, when the task cannot piggyback off the semantic or logical properties of language, or if it can, but the logical inferences required are indeterminate or too complex (and need to be augmented by other cognitive resources), the right PFC and parietal lobe systems are engaged. The same seems to be true for causal inference (see below).

To further illustrate what is meant by the claim that conceptual, logical, and causal constructs are embedded in the structure of language, let’s consider the case of simple transitive inference. There are a number of studies showing that many animals, including chimpanzees (Gillan, 1981; Boysen et al., 1993), pigeons (Delius and Siemann, 1998), and rats (Davis, 1992) can be taught to do transitive inference, or at least behave in a manner consistent with understanding transitive inference. Let’s set aside the questions of experimental design and interpretation of data (Delius and Siemann, 1998; Allen, 2006), and focus on the training regime. It takes a pigeon approximately 1600 trials to behave in a manner consistent with simple transitive inference (Delius and Siemann, 1998). How many trials does it take a human subject? If one uses the same training paradigm with human participants as one does with pigeons, it takes approximately 800 trials for a human (Acuna et al., 2002). But when transitive relations are explicitly presented, most of us understand in one or two trials. What this suggests is that we are capable of learning to do transitive inference in the same manner that a pigeon does, and we can do it in 800 trials as opposed to 1600 trials. However, the more important point is that we have certain cognitive resources—namely language—that a pigeon does not. If we utilize this system we understand simple transitive inference in one or two trials because it is built into the structure of language.

The structure of language, and indeed much of human thought, has been defined in terms of the properties of systematicity, productivity, compositionality, and inferential coherence (Fodor and Pylyshyn, 1988; Penn et al., 2008). It can be shown that a representational system with these properties needs to have discrete, nonoverlapping elements, differentiable from each other, and be reasonably precise and determinate, at least at the syntactic level (Goel, 1995). This can be referred to as syntactic determinacy.^3^ Furthermore, given such a symbol system and an inference engine one is automatically set up to make simple (local) logical and conceptual and causal connections.^4^ Once ideas are generated and represented in such a system they determine the parameters of all subsequent transformations. Each inference serves to automatically eliminate countless possibilities (reducing uncertainty). Thus the suggestion is that the structure and properties of this representational system gives rise to the left PFCs intolerance for uncertainty or indeterminacy. In the next section we focus on a complementary role of the right PFC in inhibiting these connections and keeping options open by endorsing uncertainty or indeterminacy.

## A Role for the Right PFC

It is only within the last 20 years that neuropsychologists have begun to examine reasoning and problem solving in a real-world context. Much of this research has only been recently assembled and reviewed (Goel, 2009; Marinsek et al., 2014). The result is a greater understanding of the multiple roles the right PFC plays in human inference. Goel (2009) identifies three critical roles for the right PFC, namely supporting inference in the absence of familiar conceptual content (right lateral PFC), conflict/anomaly detection (right dorsolateral PFC) and indeterminacy tolerance (right ventral lateral PFC). Marinsek et al. (2014) reviewing much of the same literature, propose that left PFC “specializes in creating hypotheses and representing causality, while the RH specializes in evaluating hypotheses and rejecting those that are implausible or inconsistent with other evidence. … The LH strives to reduce uncertainty while the RH strives to resolve inconsistency”. There is some overlap, and some interesting differences, in these two interpretations of the data. The similarities are accepting the LH role in reducing uncertainty and the RH’s role in resolving inconsistency.

One major difference is that we also see an important active role for the right PFC in *maintaining and even enhancing uncertainty or indeterminacy*. This issue is rarely addressed in the reasoning and problem-solving literatures. The normal way of viewing indeterminacy is as something undesirable that needs to be minimized and eliminated. However, it has also been argued that indeterminacy has a beneficial role to play in real-world problem solving (Goel, 1995, 2014). Here we briefly summarize the pervasiveness of indeterminacy in real-world problem solving, the importance of maintaining it (for a certain period of time), and note that maintenance of indeterminacy requires different cognitive representations and facilitates different types of transformations, and that these cognitive mechanisms are underwritten by different neural systems (Goel, 1995, 2014).

Indeterminacy results from incompleteness of information and/or non-constitutive task constraints. Incompleteness of information in each of the three components of a problem vector (start state, goal state, transformation function; Reitman, 1964), and the non-constitutive nature of the task constraints (Goel, 1995) are hallmarks of real-world problems. For example, in a simple everyday task like planning a dinner party for some guests, the start state is incompletely specified (e.g., Should it be lunch or dinner?; Should it be on a Monday or Friday? etc.). The goal state is also incompletely specified (e.g., Do I care whether they enjoy the meal? Should I take into consideration the fact that Mary is upset with John when doing the seating arrangement? etc.). And finally, the transformation function is also incompletely specified (e.g., Should the meal be catered? Should I do a potluck? If I prepare it myself should I use free range chicken? etc.). Not only are each of these three components of the problem space vector under specified, they are each negotiable. So, if I invite you to my home for dinner, and then convince you that we should instead grab a quick pizza and go to the new movie playing around the corner, and you agree, there is no sense in which I have failed the planning task. The selected solution simply lies beyond the (assumed) constraints of the original problem.

Contrast this real-world problem with a standard laboratory task, like the Tower of Hanoi (Kotovsky et al., 1985), a puzzle consisting of three pegs and several disks of varying size. The goal is to transfer the disks from one peg to another under the following three constraints: (1) only one disk may be moved at a time; (2) any disk not being currently moved must remain on the pegs; and (3) a larger disk may not be placed on a smaller disk. The Tower of Hanoi is a typical example of a well-structured problem. In such tasks the start states, goal states, and transformation functions are completely specified, and the constraints are logical or constitutive of the task. For example, if I complete the task by placing a larger disk on a smaller disk, on route, I’ve simply cheated. The former types of problems are called “ill-structured” problems while the latter are referred to as “well-structured” problems. Real-world problems have both ill-structured and well-structured components.

The basic idea is that, when confronted with a real-world problem, ranging from the ordinary (e.g., planning a dinner party), to the extraordinary (e.g., designing an affordable electric car with a range of 1000 miles), the first impulse is simply to go with what we already know. In the mundane case of planning a dinner party, the LH interpreter would start with knowledge of past parties and follow the conceptual/logical connections to the current situation and could not look beyond (suggested and assumed) constraints. It would be a prisoner of its background knowledge and beliefs. Every dinner party would be similar to the last, with little allowance for the variations and deviations that are the hallmark of human problem solving. In the case of the extraordinary problem solving situation, the problem solver may not have access to any known solutions (as there may be none), but nonetheless, there will be background beliefs and knowledge from previous experiences that will be mapped onto the problem space. The LH interpreter, by its acceptance and precise representation of this belief network and task constraints, armed with local conceptual and logical connections, would confine the problem solver to a particular space, perhaps precluding the actual solution space.

One way of circumventing such a depressing outcome is to have a complementary system to the interpreter that serves to maintain any indeterminacy that exists in the task environment (at least for a period of time), and where indeterminacy does not exist it serves to actively create it. In both the mundane and novel cases the introduction of indeterminacy is serving the function of overcoming unwarranted preconceptions based upon prior beliefs and allowing for the exploration of a broader state space.

Such a system would benefit from a representational format that supports vague, ambiguous, and indeterminate representations that have multiple interpretations that illuminate possibilities rather than concealing them (Goel, 1995). The transformations that operate on these representations may not be one of conceptual and logical connections but rather some sort of associations that allow for distal connections.^5^ Such a system is necessary to tolerate and represent the indeterminacy inherent in the real world, treating constraints and intermittent solutions as tentative, and inhibiting premature, ill-conceived inferences, until such time as the state space is explored, and alternatives considered. As the solution develops, constraints can be established and introduced into the problem space to facilitate solution refinement and detailing. This is accompanied by utilization of representational systems that are more precise, unambiguous, and determinate.

**Figure 1:**
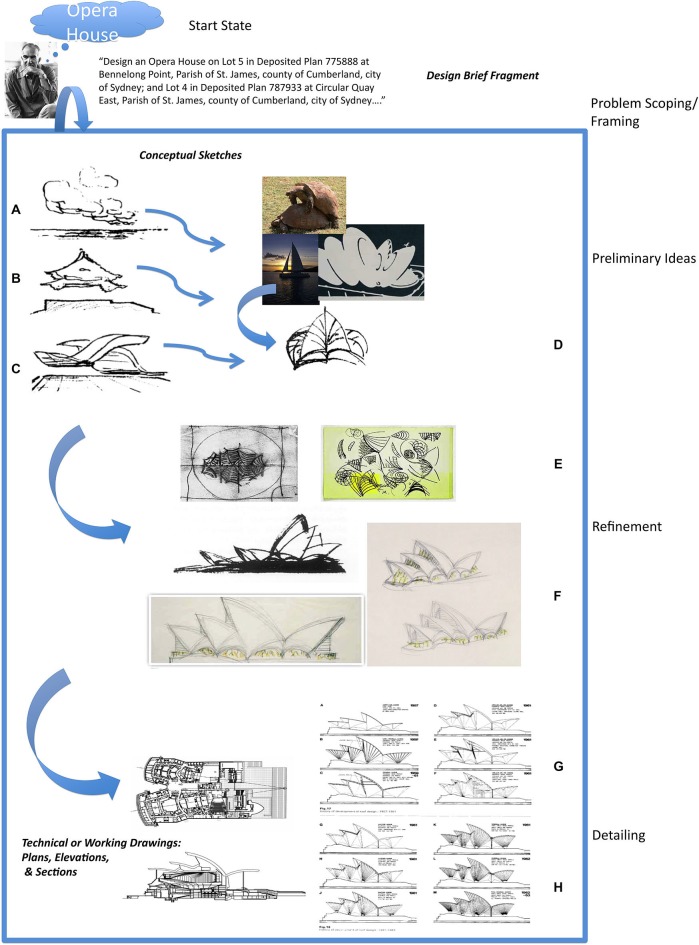
**A highly incomplete and speculative reconstruction of Utzon’s problem space for designing the Sydney Opera House**. Real world problem solving usually undergoes several distinct cognitive phases associated with different types of mental (and external) representations and transformations. In the case of architectural problem solving they can be broadly divided up into natural language, conceptual sketches, and contract documents. Their level of precision and ambiguity varies. Drawings **(A–D)** are examples of early conceptual sketches. There is often no fact of the matter as to what they represent. The “what is that” is often discovered and emerges after the drawing is made. Drawings **(E)** and **(F)** show the development of one of the ideas introduced in the conceptual sketches. The artifact is beginning to take a specific form and starting to be fleshed out. Drawings **(G)** and **(H)** are examples of technical drawings or blueprints that will form part of the contract documents. They specify the artifact in a very precise, complete, unambiguous and determinate manner. The differences between the conceptual sketches and working drawings (ostensibly both “pictorial”) are at least as great as the differences between the design brief and conceptual sketches. Reproduced with permission from Goel (2014).

The idea is illustrated in Figure 1 with an example from architectural problem solving, reproduced from Goel (2014). Figure 1 is a highly speculative and incomplete reconstruction of Jørn Utzon’s problem space for the design of the Sydney Opera House. When confronted with the design brief (problem statement) for an opera house on the Sydney Harbor, he begins by abstracting away from the particulars of the design brief, and his knowledge of opera houses, to seemingly unrelated ambiguous, amorphous, indeterminate, “meaningless”, doodles (Figures 1A–D) and reports making distal connections to the Yucatán Peninsula (Mexico), Kornborg Castle (Denmark), and the naval charts of Sydney Harbor. As he generates, selects and develops his ideas the accompanying representations become more precise and detailed (Figures 1E,F), until at the detailing stage they are unambiguous and determinate (Figures 1G,H). At this stage transformations largely involve (local) logical and conceptual connections.

This account accepts the interpreter role for the left PFC, but adds a critical, complementary role for the right PFC, and emphasizes the interaction or balance between the two hemispheres. It has been stated as the Frontal Lobe Lateralization Hypothesis in Goel (2014): “The right PFC supports abstract, vague, ambiguous, indeterminate representations of the world, while the left PFC abhors uncertainty and tries to automatically fill in the gaps with concrete, determinate, unambiguous, specific information/assumptions. The system is set up in such a way that each also tries to inhibit the other, though usually the left dominates. Successful functioning in the real world is a judicious balancing act between these two systems. Damage to either system will result in impaired real-world performance, but with different cognitive signatures. Damage to right PFC system will allow the left PFC free reign to prematurely lock onto patterns and solutions; drawing conclusions quickly and confidently, often to the detriment of the patient. Damage to the left PFC will allow the right-hemisphere system … to have more impact. If it remains totally unchecked by the LH interpreter, one would expect these patients to have enormous difficulty in articulating details and arriving at decisions”.

It is worth noting that Beeman (1993; Jung-Beeman, 2005), working with linguistic data, has developed an interesting account of hemispheric asymmetry in terms of different types of coding of linguistic representations in left and RHs. On this account, the RH utilizes “course” overlapping semantic fields to encode information, whereas the LH engages in more finer grained, less overlapping coding of information. If “fine coding” maps onto more determinate representations and “coarse coding” maps onto more indeterminate representations, then there is an overlap between these ideas, and those developed here, that requires further exploration.

In contrast, Corballis (2003), by examining the role of the RH in disambiguating and resolving stimuli in visual completion tasks, concludes that the main function of the RH is “resolving the ambiguities inherent in spatial vision.” The RH does this by creating and maintaining an analog, unambiguous, determinant, veridical representation of the world that is used to keep in check the generative proclivities of the LH. We, on the other hand, are suggesting that tunnel vision is a natural byproduct of the generative machinery of the LH that must be offset by the indeterminacy-tolerant, associative machinery of the RH.

## Evidence for Left PFC Interpreter

There is considerable evidence for the various components of the LH interpreter. The classical split brain patient studies (Gazzaniga and Smylie, 1984; Gazzaniga, 1998, 2000) and newer studies on inductive inference (Goel et al., 1997; Goel and Dolan, 2004; Reverberi et al., 2005) provide support for its role in making conceptual and semantic connections. Numerous studies provide evidence for its role in semantic and simple logical inferences (reviewed in Goel, 2007; Prado et al., 2011). Finally, studies by Wolford et al. (2000) provide evidence for its hypothesis generation function, while other studies provide evidence for its role in making simple causal connections (Gazzaniga and Smylie, 1984; Roser et al., 2005). A more comprehensive review of this literature is provided by Marinsek et al. (2014).

## Evidence for the Role of Right PFC System

Recent data suggest that right PFC is crucial in situations where the problem space: (a) is very broad, contains incomplete information and non-constitutive constraints; and/or (b) contains misleading/conflicting information. These are all hallmarks of real-world problems and robustly engage right PFC systems. For example, we have found, in an anagram task, that broadening semantic categories that words can belong to (and thus the problem space) from “make the word ‘knife’ with IKFEN”, to “make a word for a kitchen utensil with IKFEN”, to “make a word with IKFEN”, relaxes task constraints and selectively activates right PFC (Vartanian and Goel, 2005). Mental set shift tasks, like the Matchstick problems, which require the overcoming of implicit misleading cues, selectively activate right PFC in the misleading condition (Miller and Tippett, 1996; Goel and Vartanian, 2005).

Even in a classic “LH” task like logical reasoning, a patient study suggests a double dissociation such that patients with lesions to left PFC are selectively impaired in determinate trials (e.g., A > B, B > C, A > C; and A > B, B > C, C > A), while patients with lesions to right PFC are selectively impaired in indeterminate trials (e.g., A > B, A > C, B > C; Goel et al., 2007). Neuroimaging studies reveal similar results (Goel et al., 2009; Brzezicka et al., 2011).

With respect to causal inference, at least one study, involving split brain patients, shows a double dissociation between the ability of the LH and the RH to deal with simple well-structured causal relations (as in “billiard ball causation”), and more complex, indeterminate, difficult to discern and resolve spatiotemporal relationships between events (Roser et al., 2005). The LH is great at the former but the latter requires the RH.

Moreover, several studies utilizing real world design and planning tasks selectively activate right PFC systems. For example, an imaging study carried out by Kowatari et al. (2009) asked novice and experienced designers to “think about new designs” for pens. Their main finding included greater activation in right PFC than in left PFC, in the design component of the task. Furthermore, a correlational analysis using the originality scores of individuals (generated by applying a “good design award criteria” metric) and BOLD signal changes showed a correlation between the left minus right PFC BOLD signal and the originality scores, but not between left PFC or right PFC BOLD signals and originality scores *per se*. This interesting finding is consistent with our contention that interaction between right and left PFC are critical for real-world problem solving.

Gilbert et al. (2010) administered well-structured and ill-structured versions of a simple design task, involving the arrangement of furniture in a board room, to participants as they underwent MRI scanning. The well-structured version of the task contained specific constraints such as “the two tables face each other” while the ill-structured version contained more open-ended constraints such as “the room should feel spacious”. The main conclusion of the study was that the ill-structured design condition was associated with greater activation in right dorsolateral prefrontal cortex compared to the well-structured condition.

Two patient studies, involving real world design and planning tasks, have reached the same conclusion. Goel and Grafman (2000) tested a very accomplished 57 year old architect diagnosed and treated for a right frontal parasagittal meningioma, by requiring him to develop a new design for their lab space, and compared his performance to an age and education matched architect. The control architect began the task by considering abstract issues such as “circulation space” and “social/professional hierarchies”, and then used these abstract concepts to determine arrangements of walls, cubicles, etc. The patient’s sophisticated architectural knowledge base was still intact and he used it quite skillfully during the problem scoping phase to discuss various aspects of the design. However, he approached the design task at a very concrete level and just arranged furniture. He generated a quick solution, without abstracting from the particulars and exploring the space of alternatives.

In another study, Goel et al. (2013) administered a real-world planning task to neurological patients with unilateral lesions in PFC and normal controls. Patients with lesions to right PFC generated substandard solutions compared to both normal controls and patients with left PFC lesions. Examination of the underlying cognitive processes and strategies revealed that patients with lesions to right PFC approached the task at an excessively concrete level compared to normal controls, and very early locked themselves into substandard solutions. Patients with lesions to left PFC displayed a trend towards approaching the task at a more abstract level than the controls, and more fully explored solution possibilities. In contrast to both patient groups, normal controls engaged in the task at both concrete and abstract levels and easily/judiciously moved between the levels.

These data suggest differences in the capacity of left and right PFC to deal with indeterminacy. They confirm the “interpreter” role of the left PFC but also highlight the critical role of the right PFC in tolerating indeterminacy, exploring alternative possibilities, and inhibiting premature conclusions. These differences are difficult to detect in standard neuropsychological test batteries (because these tasks are largely well-structured) but they become apparent in real-world tasks which contain both well-structured and ill-structured components.

## Conclusion

The goal of this review has been to suggest that determinacy of information and constraints is one important principle of functional organization in PFC that emerges in the examination of real-world problem-solving and reasoning. Indeterminacy results from incompleteness of information and non-constitutive task constraints inherent in real-world problems. It is often viewed as something undesirable, requiring immediate elimination. This review makes the case that it is generally beneficial to retain a level of indeterminacy in many problem-solving situations to prevent tunnel vision. What is required is a controlled reduction of possibilities.

As illustrated in Figure 1, in the early phases of problem solving, one needs to step back from the particulars of the problem statement and treat the constraints as negotiable. This allows one to explore a larger space. Ambiguity and indeterminacy are desirable properties in the problem representation at this stage, and associative machinery is used to make distal connections that lie beyond the scope of local logical/conceptual inferences. As ideas are generated, merged, separated, rejected, and selected, the accompanying problem representations become more precise and detailed, and the transformations begin to take more advantage of logical and conceptual connections. This is a cyclical process usually requiring multiple iterations. The end state should be an unambiguous and determinate representation of the solution.

The data indicate that left PFC is unable to tolerate indeterminacy. I have suggested that this may be a consequence of the LH’s dominance for language. Language can be characterized as being syntactically determinate and also having the properties of generativity, systematicity, productivity, and inferential coherence. The system is essentially set up to frame the world in terms of its existing belief network and to integrate new information into the belief network by making local causal, logical, and conceptual connections^5^. As such, it is difficult for the left PFC to look beyond simple inferences from what it already knows. The right PFC, on the other hand, tolerates and represents indeterminacy by utilizing representations with very different properties than syntactic determinacy, generativity, systematicity, productivity, and inferential coherence. This allows it to bypass the tunnel vision of the left PFC and more broadly explore the state space.

Indeterminacy is offered as one basis of hemispheric lateralization. The idea emerges from the study of problem solving in real-world situations. It is not meant to account for all of the data on hemispheric lateralization. However, there are some intriguing connections between the determinacy/indeterminacy distinction and some earlier ideas mentioned in the introduction.

For instance, if we take seriously the idea that the processing capabilities of the left PFC interpreter emerge from the properties of natural language, and that the processing capabilities of the right PFC emerge from the properties of nonlinguistic types of representational systems (or at least systems that are not syntactically determinate and do not possess the properties of systematicity, productivity, generativity, and inferential coherence), we are taken back to earlier distinctions between verbal and visual-spatial systems (Ratcliff, 1979; Frost et al., 1999; Corballis, 2003).

The indeterminate/determinate distinction is more abstract and, I believe, largely independent of the verbal/visual-spatial distinction *per se*. This is illustrated in the architectural example in Figure 1 (which involves both linguistic and pictorial representations), and has been argued in detail elsewhere (Goel, 1995). However, it may be the case that the linguistic lends itself more readily to determine expressions while the nonlinguistic lends itself to indeterminate expressions (but counterexamples are easy to generate).^6^ This is why the focus should be on the determinate/indeterminate properties of representations rather than linguistic and visual-spatial *per se*.

Another traditional hemispheric distinction is in terms of local (LH dominance) vs. global (RH dominance) processing (Sergent, 1987; Robertson and Lamb, 1991). It may also be possible to make sense of this in terms of the fact that logical and conceptual inferences built into the machinery of language are typically local while associations can be arbitrarily far-reaching. Similarly, in the categorical vs. coordinate distinction (Kosslyn et al., 1989; Jager and Postma, 2003), one could argue that categories require a subject/predicate distinction built into the structure of natural language, while coordinate representations do not. These potential connections may warrant further exploration.

Finally, this is not a LH or RH account of reasoning and problem-solving. It is an account whereby each hemisphere is biased towards certain types of representational structures and processing mechanisms, distinguishable on the basis of how they deal with determinacy/indeterminacy. Successful real-world functioning requires the participation of both hemispheres.

## Conflict of Interest Statement

The author declares that the research was conducted in the absence of any commercial or financial relationships that could be construed as a potential conflict of interest.

## Footnotes

^1^Elsewhere the terms “ill-structured” and “well-structured,” and “non-notational” and “notational” representations have been used to refer to indeterminate and determinate, respectively (Goel, 1995; Goel and Grafman, 2000).

^2^There is a single study by Deglin and Kinsbourne (1996) which endorses the reverse position, where inferences involving formal logical relationships engage the left hemisphere but inferences involving conceptual or semantic connections are relegated to the right hemisphere. No other study has replicated this result.

^3^It has elsewhere been referred to as a “notational scheme” or a “discursive language” (Goodman, 1968).

^4^The meaning of “local” here can be illustrated with a logical example. Suppose one is given the axiom X • (¬A⊃B), and asked to prove the theorem (B∨ A) • X. Few people would have on hand a rule or function to directly map one onto the other. However, we can all arrive at the theorem by the sequential application of simple rules applied locally as in the following sequence: L1: X•(¬A⊃B) ⇒ (B ∨ A)•X [Given] L2: (¬A⊃B)•X    [Commutative L1] L3: (A ∨ B)•X [Material implication L2] L4: (B ∨ A)•X [Commutative L3] It is in this sense that logical transformations are local.

^5^See Fodor and Pylyshyn (1988) for a discussion of the relationship between conceptual connections and associations. Distal connections are in contrast to the local connections described in footnote 4. They are based upon various sorts of associations (perhaps serendipitous) and can be arbitrarily far reaching.

^6^For example, much of the poetry of Shelley is amorphous, multifaceted, and ambiguous. It maps onto many different interpretations. On the other hand, legal documents, scientific writings, etc. strive to be precise and unambiguous, and minimize alternative interpretations. Similarly, with nonlinguistic representations, the exploratory conceptual sketches of architects and engineers are amorphous, multifaceted, and ambiguous, but the subsequent working drawings or blueprints are precise, determinate, and unambiguous. There is a one-to-one mapping between the drawings, the world, and back again. The real issue is to what extent representational systems allow for the encoding of indeterminate states of affairs.
